# Hypertension and left ventricular geometry: Diagnosis, prognosis, and management

**DOI:** 10.17179/excli2026-9416

**Published:** 2026-06-02

**Authors:** Samantha M. Mutai, Namrata Kadambi, Mattin Moazzam, Ahmet Afsin Oktay

**Affiliations:** 1 Department of Medicine, Division of Cardiology, Rush University Medical Center, Chicago, IL, United States

**Keywords:** hypertension, left ventricular hypertrophy, cardiac remodeling, electrocardiography, echocardiography, cardiac magnetic resonance imaging, artificial intelligence

## Abstract

Hypertension remains one of the most prevalent and consequential cardiovascular risk factors worldwide and is a leading cause of heart disease. Hypertensive heart disease may manifest in alterations in left ventricular (LV) geometry, including concentric remodeling, concentric hypertrophy, and eccentric hypertrophy, representing adaptive responses to chronic pressure or volume overload that may progress to maladaptive remodeling and heart failure. LV geometric patterns, defined by LV mass and relative wall thickness, carry important diagnostic and prognostic implications independent of blood pressure levels. This review provides a comprehensive and contemporary overview of the relationship between hypertension and LV geometry. We summarize key determinants of hypertensive LV remodeling, including cumulative blood pressure exposure, sex differences, metabolic comorbidities, obesity, pericardial adiposity, and obstructive sleep apnea. We discuss current approaches to screening and diagnosis, highlighting the strengths and limitations of electrocardiography, echocardiography, and cardiac magnetic resonance imaging. We review emerging applications of artificial intelligence in electrocardiographic and echocardiographic assessment, with particular attention to their potential to improve detection, phenotypic differentiation, and prognostication. We further examine the prognostic significance of LV remodeling in hypertension and review evidence supporting regression of LV hypertrophy through intensive blood pressure control, management of comorbidities, and lifestyle interventions. Early identification and reversal of hypertensive LV remodeling may offer a critical opportunity to prevent progression to heart failure and reduce long-term cardiovascular morbidity and mortality.

See also the graphical abstract(Fig. 1).

## Abbreviations

2D: Two-Dimensional

3D: Three-Dimensional

AI: Artificial Intelligence

ARB: Angiotensin II Receptor Blocker

ARNI: Angiotensin-Receptor Blocker-Neprilysin Inhibitors

ASE: American Society of Echocardiography

AUC: Area Under the Curve

BP: Blood Pressure

CI: Confidence Interval

CMR: Cardiac Magnetic Resonance Imaging

CPAP: Continuous Positive Airway Pressure

CR: Concentric Remodeling

DASH: Dietary Approaches to Stop Hypertension

EACVI: European Association of Cardiovascular Imaging

ECG: Electrocardiogram

GLP-1: Glucagon-Like Peptide-1

HF: Heart Failure

HFpEF: Heart Failure with Preserved Ejection Fraction

HR: Hazard Ratio

LV: Left Ventricle

LVEF: Left Ventricular Ejection Fraction

LVH: Left Ventricular Hypertrophy

cLVH: Concentric Left Ventricular Hypertrophy

eLVH: Eccentric Left Ventricular Hypertrophy

LVM: Left Ventricular Mass

MESA: Multiethnic Study of Atherosclerosis

NT-proBNP: Amino-Terminal Pro-B-type Natriuretic Peptide

OR: Odds Ratio

OSA: Obstructive Sleep Apnea

PA: Primary Aldosteronism

RCT: Randomized Controlled Trial

RWT: Relative Wall Thickness

T2DM: Type 2 Diabetes Mellitus

## Introduction

Hypertension is a widespread cardiovascular disease risk factor, with a global prevalence of 32-34 % among adults aged 30-79 (approximately 1.28 billion people in 2019), as well as a substantial clinical impact on cardiovascular morbidity and mortality (Zhou et al., 2021[67]). Hypertensive heart disease is common and has various multisystemic manifestations. One of the most common cardiovascular manifestations of hypertension is left ventricular (LV) hypertrophy (LVH). The prevalence of LVH among individuals with hypertension varies by study population and imaging modality. (Park et al., 2022[48]; Cuspidi et al., 2012[16]).

To understand the clinical implications of the relationship between hypertension and LVH, it is essential to distinguish normal LV geometry from hypertension-induced LV remodeling, which is commonly grouped into three geometric patterns: concentric remodeling (CR), concentric LVH (cLVH), and eccentric LVH (eLVH). These patterns are distinguished by LV mass (LVM) and relative LV wall thickness (RWT), a proportion of wall thickness as compared to the cavity size, as determined by echocardiographic measurements and various geometric formulas described later in this review (Figure 2(Fig. 2)) (Lang et al., 2015[38]).

CR generally occurs as an early adaptation to reduce myocardial strain in pressure overload states, such as hypertension, where increased wall stress leads to an increase in RWT (> 0.42) and a decrease in chamber size, while maintaining a normal LVM. Conventionally, cLVH develops when, over time, a higher systolic wall stress leads to the compensatory addition of new sarcomeres in parallel, increasing the cross-sectional area of each myocyte and both RWT (> 0.42) and LVM. In contrast, eLVH occurs when increased wall stress during diastole, as seen in conditions of volume overload such as valvular disease, causes the addition of new sarcomeres in series, increasing the length of each myocyte as well as LV cavity size and LVM, with a normal RWT (≤ 0.42) (Marwick et al., 2015[45]). These changes come from adaptive responses to stressors that eventually become maladaptive and can lead to poor outcomes.

A post-hoc analysis of the Antihypertensive and Lipid-Lowering Treatment to Prevent Heart Attack Study (ALLHAT) trial, including 33,357 patients with hypertension, revealed that both the presence of LVH by electrocardiogram (ECG) diagnosis and a higher degree of ECG LVH by Cornell voltage were associated with a significantly higher risk of death, coronary heart disease, nonfatal myocardial infarction, stroke, angina, and heart failure (HF) over a six-year follow-up period. These associations were observed independent of comorbidities or antihypertensive treatment. Furthermore, the persistence or development of LVH, as indicated by ECG, while on antihypertensive therapy, was associated with a higher rate of cardiovascular events, regardless of actual blood pressure (BP) (Bang et al., 2017[4]).

A large, multicenter prospective study from the early 2000s showed that patients with a baseline normal LV ejection fraction (LVEF) who had echocardiographic LVH were more likely to develop reduced LVEF over a 5-year follow-up period than those without LVH, with a risk ratio of 2.2. This difference persisted even in the absence of myocardial infarction, which is considered the primary mechanism underlying a decline in LVEF. Those with an eccentric pattern of hypertrophy had a stronger association with depressed LVEF (relative risk 2.3) than other LV geometric patterns (Drazner et al., 2004[24]).

This article provides a comprehensive, up-to-date review of the relationship between hypertension and LV geometry, highlighting the detection of LV abnormalities, the use of artificial intelligence in diagnosis and prognostication, and current and emerging management strategies for LVH (Figure 1(Fig. 1)).

## Determinants of Left Ventricular Hypertrophy

Geometric patterns of LV remodeling are associated with various risk factors, including age, race, sex, and duration and severity of hypertension (Vasan et al., 2022[58]). LV remodeling has also been associated with multiple other cardiovascular risk factors, including chronic kidney disease, obesity, valvular heart disease, ischemic heart disease, and type 2 diabetes mellitus (T2DM).

A community-based cross-sectional study of asymptomatic patients aged 60-85 with longstanding hypertension (median duration of hypertension diagnosis of 10 years) revealed an overall prevalence of LVH as 44 %. The prevalence increased with age, reaching 60 % in individuals ≥ 75 years. As expected, the study also revealed that ECG sensitivity for detecting LVH was lower than that of echocardiography (de Hartog-Keyzer et al., 2020[19]).

### Exposure to high blood pressure

There is a strong association between cumulative BP burden and the development of LVH. In a prospective cohort study of 6,105 adults free of baseline cardiovascular disease followed for a median of 14 years, hypertension was associated with more than a two-fold increased odds of ECG-LVH (adjusted odds ratio [OR] 2.45, 95 % confidence interval [CI] 1.83-3.30, *p* < 0.001), with a stepwise increase in LVH prevalence across higher systolic BP categories (Cao et al., 2019[11]). Importantly, longitudinal analyses from the CARDIA study further clarified the role of duration and cumulative exposure, demonstrating that greater long-term exposure to elevated BP beginning in young adulthood is associated with increased LVM and a higher prevalence of LVH in midlife, independent of current BP levels (Kishi et al., 2015[35]). Additional population-based studies similarly support these concepts, with evidence linking higher BP severity and adverse BP trajectories over time to increased LVM and LVH (Lai et al., 2014[37]; Zhou et al., 2023[68]), as well as studies demonstrating that prolonged duration of elevated BP exposure is independently associated with hypertensive cardiac remodeling (Cuspidi et al., 2019[15]; Masrouri et al., 2025[46]).

As expected, BP control significantly impacts LV remodeling in individuals with hypertension. Soliman et al. evaluated the effect of intensive (BP goal of < 120 mmHg) versus standard (BP goal of < 140 mmHg) BP control on ECG-LVH among 8,164 participants of the Systolic Blood Pressure Intervention Trial (SPRINT). The analysis demonstrated that, compared with standard BP, intensive BP control was associated with a lower risk of LVH development (hazard ratio [HR] 0.54, 95 % CI 0.43-0.68, *p* < 0.001) and a higher likelihood of LVH regression (HR 1.66, 95 % CI 1.31-2.11, *p* < 0.001) (Soliman et al., 2017[54]).

### Sex-related differences

Sex is a primary determinant of LV remodeling in patients with hypertension. A prospective registry of 6,427 subjects evaluated the interaction between sex and echocardiographically determined LV remodeling over a mean follow-up of 6.1 years (Canciello et al., 2024[10]). At baseline, the prevalence of LVH was higher in female subjects than in male subjects (47 % vs. 23 %), including both eLVH (40 % vs. 18 %) and cLVH (7 % vs. 5 %). During follow-up, female subjects had a significantly higher risk of progression to pathologic LV geometry than male subjects, with an adjusted OR of 2.50 (95 % CI 2.25-2.77, *p* < 0.001). Longer history of hypertension, poor BP control, and treatment with more antihypertensive drugs were also associated with a higher risk of pathologic LV geometry at follow-up (Canciello et al., 2024[10]). These differences may help inform risk stratification, as well as support closer surveillance for adverse remodeling in females with longstanding or poorly controlled hypertension.

Beyond general sex differences, reproductive history may also influence susceptibility to LVH. Hauge et. al. demonstrated in a cohort study of 679 women aged 40-55 years (a median duration of 15 years after pregnancy) that the prevalence of LVH was higher in women with a history of preeclampsia (OR 1.62, 95 % CI 1.07-2.46, *p* = 0.024) compared to the general population, regardless of the presence of chronic hypertension (Hauge et al., 2025[28]).

### Impact of comorbidities

Metabolic disorders such as T2DM and obesity further amplify the risk of LVH. An analysis of 5,741 participants in the Framingham Heart Study demonstrated that T2DM and overweight/obesity were independently associated with LV geometric abnormalities, with evidence of synergistic effects among T2DM, body mass index (BMI) category, and hypertension. Together, these data suggest that clustering of metabolic and hemodynamic risk factors may accelerate maladaptive LV structural changes and increase susceptibility to HF (von Jeinsen et al., 2020[61]). A cross-sectional study of 2,912 diabetic patients similarly demonstrated a higher prevalence of LVH among those with diabetic microvascular complications, particularly retinopathy and diabetic kidney disease (Liu et al., 2025[43]). This association further supports a link between systemic microvascular injury and adverse cardiac remodeling.

The impact of general and abdominal obesity on LV structure and function has also been examined. In a cross-sectional study of 971 Chinese individuals with hypertension but without overt cardiovascular disease, patients with general obesity, defined as a BMI ≥ 28 kg/m2, had higher LV end-diastolic volume, LVM, and LV filling pressures, as assessed by left atrial volume and the septal E/e' ratio. Those with abdominal obesity, defined as a waist circumference ≥ 90 cm for men and ≥ 85 cm for women, were associated with impaired LV relaxation as determined by septal e' velocity (Cai et al., 2021[9]).

Beyond obesity, the distribution of adipose tissue may specifically contribute to hypertensive remodeling. In a large population-based study including 42,598 participants from the UK Biobank CMR study, higher pericardial adiposity was independently associated with adverse LV structure and function after adjustment for anthropometric measures of obesity and visceral adiposity. Specifically, greater pericardial adipose tissue was associated with increased LV wall thickness, higher LVM, a more concentric remodeling pattern (higher LVM/LV end-diastolic volume), and impaired systolic performance. Mediation analyses demonstrated that these associations were not fully accounted for by vascular risk factors, inflammatory markers, or lipid profiles, suggesting a potential direct contribution of pericardial adiposity to remodeling phenotypes, possibly through local paracrine or mechanical effects (Ardissino et al., 2022[2]).

In addition to these factors, sleep-related disorders, particularly obstructive sleep apnea (OSA), have emerged as important contributors to the development and progression of LVH. OSA is characterized by recurrent upper airway collapse during sleep, and the resulting pathophysiologic changes lead to hypertension and increased LV afterload, promoting maladaptive cardiac remodeling (Shiina, 2024[53]).

A 2020 meta-analysis demonstrated a significant association between OSA and LVH, with an OR of 1.70 (95 % CI 1.44-2.00, p < 0.001) (Cuspidi et al., 2020[17]). This association was even stronger for patients with more severe or uncontrolled hypertension. In an observational study, OSA was present in over 51 % of adults with hypertension and was independently associated with LVH among patients with resistant hypertension, with an OR of 4.31 (95 % CI:1.14-16.34; *p* = 0.032) (Cabrini et al., 2023[8]). These findings suggest that OSA contributes to hypertension-mediated organ damage and cardiac remodeling.

## Screening and Diagnosis of LVH in Patients with Hypertension

In clinical practice, a variety of diagnostic modalities, such as ECG, 2D and 3D transthoracic echocardiography, and cardiac magnetic resonance imaging (CMR), can be used to diagnose LVH. According to the most recent guidelines published in 2025, resting ECG is recommended as part of the initial evaluation of adults with hypertension to assess for cardiac implications such as LVH (Writing Committee Members, 2025[62]). Routine transthoracic echocardiography screening is not recommended for asymptomatic individuals with uncomplicated hypertension. However, it may be appropriate when clinical suspicion of structural disease or HF exists, or when results would influence management decisions.

### Electrocardiography

ECG is a first-line diagnostic modality for detecting LVH in hypertensive patients. There are various ECG criteria for diagnosing LVH (Figure 3(Fig. 3)). The most well-known are the Sokolow-Lyon criteria (SV1 + RV5/RV6 ≥ 35 mm) and the Cornell criteria (RaVL + SV3 > 28 mm for male subjects and > 20 mm for female subjects). While ECG is a low-cost, quick, non-invasive, and readily available test, its sensitivity for the detection of LVH is relatively low (14-47 %) when compared to echocardiography as the gold standard, while it generally has a higher specificity (75-92 %) (Bressman et al., 2020[6]; Bult et al., 2024[7]; de Hartog-Keyzer et al., 2020[19]). A retrospective study of a diverse population of 13,960 subjects demonstrated that sensitivity was higher in patients with lower BMI, male patients, and non-Hispanic white patients compared to other racial groups. Specificity was higher among female patients and those with a BMI > 30 (Bressman et al., 2020[6]), suggesting that greater adiposity may interfere with ECG detection of LVH.

A study comparing various ECG voltage criteria for the diagnosis of LVH revealed that the Cornell voltage criteria had a higher sensitivity (21 %) and specificity (92 %) than the Sokolow-Lyon criteria (14 % and 90 %, respectively) (Bult et al., 2024[7]). Most recently, in 2017, novel voltage criteria, the Peguero-Lo-Presti criteria (deepest S wave in any lead [SD] + S wave in lead V4 [SV4] ≥ 28 mm for males and ≥ 23 mm for females), were introduced as another valuable tool for detecting LVH. A meta-analysis of four studies found that the Peguero-Lo-Presti criteria had higher sensitivity (56 %) and similar specificity (90 %) compared with the Cornell (36 % and 93 %, respectively) and Sokolow-Lyon (24 % and 97 %, respectively) criteria (Yu et al., 2021[65]).

### Echocardiography

While ECG findings help detect LVH, TTE is the most commonly used imaging modality for detailed evaluation of LV geometry. A retrospective study of 1,743 patients enrolled in the Korean Hypertension Cohort comparing TTE and ECG showed a higher risk of major adverse cardiovascular events and death in subjects with LVH diagnosed by ECG alone (adjusted HR 1.69, 95 % CI 1.22-2.35, *p* = 0.001) or ECG and TTE (adjusted HR 1.87, 95 % CI, 1.18 - 2.94, *p* = 0.007) than subjects with LVH diagnosed by TTE alone (adjusted HR 1.54, 95 % CI 1.16-2.05, *p* = 0.002), when compared to subjects without LVH, suggesting poorer outcomes when LVH is detectable by ECG. These results indicate that TTE is a more effective screening tool, potentially helping detect LVH at an earlier stage of the disease, enabling earlier intervention to prevent adverse outcomes (Park et al., 2022[48]).

As outlined above, the various geometric patterns of LVH are distinguished by LVM and relative wall thickness. The most recent guidelines from the American Society of Echocardiography (ASE) and the European Association of Cardiovascular Imaging (EACVI) describe multiple standardized methods for quantifying these aspects of LV geometry using validated linear and two-dimensional (2D) measurements (Figure 4(Fig. 4)) (Lang et al., 2015[38]).

Relative wall thickness is calculated as (2×PWTd)/LVIDd, where PWTd is the posterior wall thickness at end-diastole, and LVIDd is the LV's widest internal diameter at end-diastole, obtained from the parasternal long-axis view using 2D images. These linear methods remain the standard for quantifying wall thickness.

LVM is also primarily calculated from linear dimensions taken from 2D-guided (preferred) or M-mode measurements using the ASE/EACVI-recommended formula LV mass = 0.8×{1.04[(LVIDd+PWTd+SWTd)3−(LVIDd)3]}+0.6 g, where SWTd is the septal wall thickness at end diastole. This formula is derived by subtracting the LV cavity volume from the volume within the LV epicardium, yielding the LV muscle volume, which is then converted to LVM using the myocardial density. This calculation is prone to error because it relies on cubed linear measurements and assumptions of normal LV shape, and thus is limited when the ventricular shape is distorted, such as in regional wall scar or asymmetric hypertrophy (Lang et al., 2015[38]).

Alternative approaches to LVM measurement include the area-length method and truncated-ellipsoid models derived from 2D imaging, which reduce the need for geometric assumptions. Three-dimensional (3D) echocardiography can also be used to calculate LVM and offers the advantage of directly measuring myocardial volume without geometric assumptions, making it particularly useful in abnormally shaped ventricles or asymmetric hypertrophy; however, the current evidence base is insufficient to establish normal reference values, limiting its routine clinical application. As a result, linear methods remain the most widely used in clinical practice. Measurements of LVM are also indexed to body size, typically body surface area, to ensure that comparisons across individuals are meaningful. These measurement approaches and indexing recommendations are detailed in the ASE/EACVI chamber quantification guidelines (Lang et al., 2015[38]).

### Cardiac magnetic resonance imaging

CMR is another modality that helps characterize LVH in patients with hypertension. CMR provides accurate measurements of LV wall thickness, chamber size, and hypertrophy distribution to assess LV geometry. CMR is not routinely used in the evaluation of patients with hypertensive heart disease. However, it can be beneficial for myocardial tissue characterization, including the detection of diffuse fibrosis and scar, and offers incremental value beyond echocardiography. A study of patients with hypertensive heart disease demonstrated that CMR can identify diffuse myocardial fibrosis and subclinical dysfunction, providing insight into early myocardial remodeling that may not be apparent on conventional echocardiography (Rodrigues et al., 2016[50]). The study further demonstrated distinct tissue and functional profiles across LVH phenotypes in hypertensive patients, suggesting a potential role for CMR in phenotypic differentiation beyond geometric measures alone.

In addition, CMR facilitates the identification of non-hypertensive causes of LV wall thickening, including infiltrative disorders such as amyloidosis, hemochromatosis, Fabry disease, and Friedrich ataxia. Accordingly, the Society for Cardiovascular Magnetic Resonance consensus document supports the use of CMR in the evaluation of myocardial disease when LV wall thickening is unexplained by hypertension severity or when infiltrative cardiomyopathy is suspected (Leiner et al., 2020[39]).

Importantly, CMR is not affected by common limitations of echocardiography, including poor acoustic windows due to body habitus and the geometric assumptions inherent in calculating LVM and wall thickness from linear measurements. However, the use of CMR is limited by its reduced availability, longer examination and processing times, lower patient tolerability, and higher costs than ECG and echocardiography (Lewis and Rider, 2020[40]).

## Role of Artificial Intelligence in LVH Detection and Differentiation

Artificial intelligence (AI) is rapidly transforming cardiology by enhancing diagnosis, clinical management, and risk stratification. An extensive list of AI algorithms can now accurately detect a wide range of ECG abnormalities. A growing literature has evaluated the role of AI in detecting and differentiating LVH using ECG and echocardiographic images.

### Electrocardiogram AI

ECG AI has advanced rapidly, enabling automated interpretation and detection of subtle ECG patterns that are often unapparent to human readers. Deep learning models have demonstrated high accuracy in identifying LVH and other structural heart diseases. Studies have shown that ECG AI can improve early detection of LVH. However, challenges remain regarding generalizability, bias, and regulatory approval. The American Heart Association highlights the transformative potential of ECG AI for scalable, precise, and earlier detection of cardiovascular disease, while emphasizing the need for rigorous validation and equity in deployment (Armoundas et al., 2024[3]).

AI-based models significantly outperform traditional ECG measures in detecting LVH. A meta-analysis by Suchal et al., which compiled data from nine studies totaling over 31,600 patients in testing and over 100,000 in training sets, evaluated the accuracy of AI-ECG models compared with anatomic evaluation methods, such as echocardiography or MRI. The study found that AI models notably exceeded conventional Sokolow-Lyon and Cornell criteria in diagnostic accuracy for LVH detection (Suchal et al, 2025[57]). Liu et al. also supported the previous study by developing a deep learning model to detect LVH from 12-lead ECGs, using echocardiography as the reference standard. The study included 28,745 adults aged 20-60 years. In the primary cohort, the AI model achieved an area under the curve (AUC) of 0.89 with 90.3 % sensitivity and 69.3 % specificity, substantially outperforming cardiologists using conventional criteria (AUC 0.64) (Liu et al., 2022[41]).

Recent studies have expanded the application of AI beyond binary LVH detection to differentiate distinct structural patterns in hypertension mediated LVH phenotypes using ECG data. One such study analyzed 20,439 hypertensive participants from the UK Biobank, in which Naderi et al. developed machine learning models that integrated ECG-derived biomarkers and clinical variables to classify four CMR-defined phenotypes: normal LV geometry, CR, eLVH, and cLVH. Among the classification models, the support vector machine was the most notable, achieving an accuracy of 0.79, sensitivity of 0.59, specificity of 0.87, and AUC of 0.69 on the UK Biobank test set. External validation in an independent cohort from the Study of Health in Pomerania showed similar performance (accuracy 0.75, sensitivity 0.51, specificity 0.85, precision 0.63, F1 score 0.56, and AUC 0.65), supporting the model's reproducibility (Naderi et al., 2025[47]). Model performance was superior for the classification of eLVH and cLVH (AUC of 0.86 and 0.72, respectively) compared with normal LV geometry and CR phenotypes (AUC of 0.65 and 0.64, respectively). In addition to phenotypic differentiation, the investigators evaluated clinical outcomes. They found that patients with ECG-predicted eLVH had over 3.4 times the risk of developing HF compared with patients with normal LV geometry (HR 3.42, 95 % CI 1.06-9.86) (Naderi et al., 2025[47]). These results demonstrate the potential role of ECG-based machine learning models in differentiating hypertension-mediated LVH and their value in predicting long‑term outcomes.

### Echocardiogram AI

AI models have been developed to automate various aspects of echocardiographic evaluation, including quantification, pathology detection, and report generation. A study by Holste et al. showcased how echocardiogram AI has evolved from single-task, single-view models to comprehensive multitask systems capable of automating all aspects of echocardiographic interpretation. The multitask system AI model, PanEcho, demonstrated high diagnostic accuracy (median AUC ≈ 0.91) across multiple diagnostic and quantitative tasks, including estimation of LV ejection fraction, evaluation of LV wall thickness, and detection of valvular and chamber dysfunction (Holste et al., 2025[30]). PanEcho maintained robust performance across diverse patient populations, imaging protocols, and point-of-care settings, potentially accelerating workflows and reducing reliance on expert readers.

In a large-scale study by Duffy et al. (2022[25]), deep learning algorithms were applied to 2D transthoracic echocardiographic videos, specifically parasternal long-axis and apical four-chamber views, to automate the measurement of LV wall thickness and chamber dimensions. The model was trained and validated on a cohort of 23,745 patients from Stanford Health Care and Cedars-Sinai Medical Center, with a mean age of approximately 62 years and a balanced sex distribution (around 54-62 % female, depending on subgroup). The AI system demonstrated strong performance, achieving a mean absolute error of approximately 1.2 mm in wall thickness measurements, and was faster and more consistent than manual physician assessments across the dataset. Beyond measurement, the model was trained to differentiate hypertrophic cardiomyopathy and cardiac amyloidosis, achieving AUCs of 0.98 and 0.83, respectively, with strong external validation across multiple centers and populations (Duffy et al., 2022[25]).

Other studies have explored AI-based differentiation of hypertrophy etiologies, including hypertensive LVH. For example, an echocardiography-based deep learning model reported an AUC of 0.88 for accurately classifying hypertensive heart disease, alongside other causes of LVH, suggesting that AI holds promise in this area as well (Yu et al., 2022[64]).

Collectively, the literature supports integrating AI into clinical workflows for the detection and differentiation of LVH, with the strongest evidence for echocardiographic and ECG-based models. However, these studies also highlight the need for additional research to demonstrate the efficacy and accuracy of AI models in real-world settings before they can be confidently integrated into clinical practice.

## Prognostic Significance of LV Remodeling in Hypertension

LV remodeling is a short-term compensatory adaptation that can become harmful over time. Adaptive changes progress to maladaptive processes when they cause increased stiffness and myocardial oxygen demand, for example, leading to impaired diastolic and systolic function and increased wall stress, which contribute to adverse cardiovascular outcomes related to hypertension. Hypertensive LVH is a significant prognostic marker for an increased risk of HF and cardiovascular mortality (Verdecchia et al., 1996[60]; Zhou et al., 2023[68]).

An analysis from the Framingham Heart Study found that the incidence of HF over 20 years was significantly higher in individuals with cLVH (13.4 %) or eLVH (15.3 %) than in those with normal geometry. The risk of HF, even after adjustment for comorbidities and myocardial infarction, persisted (Velagaleti et al., 2013[59]). Importantly, the risk of incident HF increased with the severity and complexity of LV remodeling patterns (Zile et al., 2014[69]).

An analysis of 4,979 participants of the Multi-ethnic Study of Atherosclerosis (MESA) study showed a significantly higher risk of all-cause mortality with eLVH (HR 2.58) and cLVH (HR 1.84), as well as increased incidence of HF (HR 3.78 and 3.09, respectively) over a 15-year follow-up period, as compared to those with normal LV geometry. cLVH was associated with increased incidence of myocardial infarction and coronary heart disease, while eLVH was not. cLVH was also associated with higher serum creatinine, urine albumin-to-creatinine ratio, and fasting glucose levels than eLVH and normal LV geometry. Both cLVH and eLVH were associated with higher levels of inflammatory markers, including C-reactive protein, interleukin-2, interleukin-6, and tumor necrosis factor-alpha, compared with normal LV geometry (Ha et al., 2022[27]).

While it has been shown that LVH and hypertension are both risk factors for developing HF, it is also important to understand which patients with LVH and hypertension are at the highest risk for progressing to HF. Unsurprisingly, more severe LVH is associated with a higher risk of progression to HF. Specifically, malignant LVH, or LVH combined with an elevated cardiac biomarker reflecting either injury (high-sensitivity cardiac troponin T) or strain (amino-terminal pro-B-type natriuretic peptide [NT-proBNP]), is predictive of progression to asymptomatic LV dysfunction, HF (specifically with reduced ejection fraction), and cardiovascular death (Peters et al., 2018[49]). Additional risk factors include left atrial enlargement, as one retrospective study showed that in patients with LVH and hypertension, left atrial enlargement was the best predictor of HF with preserved EF (HFpEF) development (Kassab et al., 2025[32]). Another study of more than 4,000 patients with hypertension showed that LVH and left atrial abnormalities on ECG were associated with increased mortality over 14 years compared to hypertensive patients without these abnormalities. Furthermore, concomitant LVH and left atrial abnormalities were associated with a higher risk than either abnormality alone (Ahmad et al., 2019[1]).

A large multicohort study by Dieden et al. using population-based samples showed that asymptomatic LVH and diastolic dysfunction were independently associated with an increased risk of incident HF over long-term follow-up. Notably, LVH was associated with more than double the increased risk of developing HF after adjustment for traditional cardiovascular risk factors (HR 2.05; 95 % CI 1.38-3.03; *p* = 3.4 × 10^−4^) (Dieden et al., 2025[22]). These findings underscore the prognostic relevance and significance of early hypertensive cardiac remodeling. The study also highlighted that LVH and diastolic dysfunction frequently coexisted (OR 1.76; 95 % CI 1.34-2.28), suggesting that the pathophysiological pathways may overlap and reinforce each other. Furthermore, the authors examined several biomarkers and found that Plasma NT-proBNP was the only biomarker consistently associated with both LVH and diastolic dysfunction across cohorts. These findings support the use of the biomarker Plasma NT-proBNP as an indicator of subclinical cardiac end-organ damage and highlight its prognostic value in the presence of hypertensive structural cardiac changes (Dieden et al., 2025[22]; Cleland et al., 2022[13]).

In summary, LV remodeling in hypertension is a powerful predictor of HF, strongly indicating its role in risk stratification and management in hypertensive patients.

## Clinical Management for Prevention and Reversal of Hypertensive LVH

Because of the association between LVH and HF, preventing LVH and the progression from LVH to clinical HF is crucial to improving patient outcomes and reducing mortality.

### Blood pressure control

One strategy shown to be effective in preventing this transition from LVH to HF is aggressive BP control. Major randomized controlled trials (RCTs) have confirmed the benefit of aggressive BP control in reducing fatal and nonfatal cardiovascular events and all-cause mortality (SPRINT Research Group, 2015[56]; Zhang et al., 2021[66]).

LVH regression with antihypertensive therapy has been associated with improved prognosis, underscoring the importance of early detection paired with aggressive hypertension management to prevent or reverse remodeling (Yildiz et al., 2020[63]; Đorđević et al., 2024[23]). For example, a study by Devereux et al. showed that Losartan, an angiotensin II receptor blocker (ARB), can regress LVH and improve prognosis. Importantly, this regression was associated with improved cardiovascular outcomes, including reduced stroke risk and cardiovascular morbidity and mortality (Devereux et al., 2004[21]).

Additional support of the prognostic significance of LVH regression is provided by a longitudinal cohort study that evaluated 1,847 hypertensive patients who underwent serial echocardiographic assessment at baseline and during antihypertensive treatment. The study found that baseline LVH prevalence did not differ significantly between patients younger than 65 years and those aged 65 years or older (age < 65 years: 42.6 %; age ≥ 65 years: 45.7 %; *p* = 0.187). However, LVH regression occurred more frequently in the younger group (36.4 % vs. 27.5 %; *p* = 0.008). Reductions in systolic BP were also notably associated with significant decreases in LVM index in the younger group (β = 0.425; *p* < 0.001), but not in the older group (β = 0.044; *p* = 0.308) (Chu et al., 2024[12]). Despite this age-related difference, regression of LVH itself was consistently associated with a lower risk of cardiovascular death and hospitalization for HF in both age groups. Overall, the data indicate that while age can influence the effect of BP reduction on achieving LVH regression, the presence of LVH regression itself has significant prognostic benefit across age groups. Specific antihypertensive therapies, such as ARBs, have been shown to improve prognosis by promoting LVH regression, underscoring their role in the treatment of LV remodeling to prevent HF.

Comparisons of different antihypertensive agents have shown that angiotensin-receptor blocker-neprilysin inhibitors (ARNI) may achieve a greater reduction in LVM than ARBs alone. A double-blind, multicenter RCT randomized 114 patients to either sacubitril-valsartan or olmesartan and found that LVM index decreased to a greater extent in the sacubitril-valsartan group than in the olmesartan group from baseline to 12 and 52 weeks of treatment. These differences remained significant even after adjustment for systolic BP, suggesting that ARNI have beneficial effects on LVM independent of BP-lowering impacts (Schmieder et al., 2017[52]). A detailed comparison of all antihypertensive agents for the prevention of LVH and HF is beyond the scope of this review.

A prospective cohort study in 2022 showed that, among 2,656 patients, higher long-term BP was an independent risk factor for both LVH and left atrial enlargement (Kim et al., 2022[33]). In addition to daytime and clinic BP, it may be helpful to monitor patients' BP in the ambulatory setting, particularly at night. A 2022 Korean prospective cohort study showed that isolated nocturnal hypertension was common in the general population, even in asymptomatic patients without known daytime hypertension. In this study, nocturnal hypertension was associated with greater arterial stiffness, LVH, and diastolic dysfunction than in normotensive patients, suggesting that ambulatory BP monitoring, even at night, could help prevent HF and cardiovascular disease in patients who would not otherwise be considered high risk (Kim et al., 2022[34]).

### Diet and exercise

Non-pharmacologic lifestyle interventions, such as diet, exercise, and weight management, also affect BP and LV geometry. A 2002 RCT in 133 overweight, sedentary adults with mild hypertension randomized participants to exercise training, exercise plus dietary weight-loss counseling, or no intervention and measured LVM and BP at a 6-month follow-up. Both interventions resulted in a significant decrease in BP compared with no intervention. However, only the exercise plus dietary weight management group showed significant decreases in LVM and significant regression of CR (Hinderliter et al., 2002[29]).

A 2021 RCT of 140 patients with resistant hypertension examined whether a four-month intensive lifestyle intervention program, including dietary counseling, behavioral weight management, and exercise, resulted in better BP control and lower LVM than the control group, which received only a single lifestyle counseling session. The intensive intervention group showed a significant improvement in systolic BP but no significant change in LVM (Blumenthal et al., 2021[5]).

### Weight loss

Furthermore, weight loss has also been shown to improve LV geometry and function. Obesity promotes LV concentric geometry, especially in middle-aged individuals, and weight loss, through calorie restriction and bariatric surgery, is associated with improved LV geometry and clinical, morphological, and functional improvements in LVH and HF (de Simone et al., 2016[20]; daSilva-deAbreu et al., 2021[18]). A 2018 retrospective cohort study showed that in obese, hypertensive patients, weight loss promoted a significant decrease in LVM independent of BP control. All 1,546 patients in this study received general dietary recommendations based on the Dietary Approaches to Stop Hypertension (DASH) diet, and LVM was compared between patients who achieved greater than 5 % body weight reduction and those who did not, over a mean follow-up period of 50 months. Patients who achieved weight loss had significantly lower LVM than those who did not. (Lønnebakken et al., 2019[44]).

## Management of Comorbidities

### Diabetes and Obesity

Coexisting T2DM and hypertension markedly contribute to cardiac remodeling. One study using comprehensive echocardiography in more than 1,000 patients with and without T2DM and without known heart disease found that T2DM was associated with increased LV wall thickness and decreased LV internal diameter, and that more pronounced cardiac structural changes were observed with longer duration of T2DM (Jørgensen et al., 2016[31]). The LIFE study showed that among patients with LVH who were all treated for hypertension over 4.8 years, those with T2DM had more residual LVH and less improvement in systolic LV function by echocardiography after the hypertensive treatment, suggesting that T2DM is an independent risk factor for LVH and should be a target for treatment and reduction of HF risk (Gerdts et al., 2009[26]).

Glucagon-like peptide-1 (GLP-1) agonists are a class of medications increasingly used for weight loss. In 2023, an RCT randomized 529 patients with HFpEF and obesity to semaglutide or placebo for 1 year. The study found that treatment with semaglutide led to larger reductions in symptoms and physical limitations, greater improvements in exercise function, and greater weight loss than placebo (Kosiborod et al., 2023[36]). Studies have also examined the effects of these drugs on cardiac function and geometry. A 2014 meta-analysis of 8 RCTs and prospective studies examined how GLP-1 agonists affect cardiac function and remodeling. They found that GLP-1 agonists significantly improve LVEF compared to controls, with the most benefit in HF patients. However, they found no significant changes in LV end-diastolic or end-systolic volumes. This finding suggests that GLP-1 agonists have cardioprotective effects, but the study did not demonstrate an improvement in LV size (Liu et al., 2014[42]). Another RCT in 491 patients treated with either semaglutide or placebo showed that over 52 weeks, semaglutide significantly attenuated left atrial remodeling progression and reduced RV enlargement, but did not significantly affect LV dimensions, mass, or systolic function (Solomon et al., 2024[55]). Overall, further research is needed to determine the specific cardiac remodeling benefits of GLP-1 agonists.

### Obstructive sleep apnea

Patients with OSA are at increased risk of developing LVH and HF. The impact of OSA treatment on LV remodeling has been evaluated in only a few interventional studies. A prospective interventional study of 25 patients with severe OSA demonstrated that 6 months of continuous positive airway pressure (CPAP) therapy reduced LVH, while left and right atrial enlargement remained unchanged. (Cloward et al., 2003[14]).

### Hyperaldosteronism

Another comorbid condition whose treatment has implications for BP and LVH is primary aldosteronism (PA). A 2013 RCT assigned 180 patients with PA to either adrenalectomy or medical therapy with mineralocorticoid receptor antagonists, and examined BP and LVH before and after intervention, and also compared them to 143 patients with optimally treated primary hypertension. There was no significant difference in BP and LVM change between the medically treated PA and adrenalectomy groups, but adrenalectomy patients did require significantly less BP medication. However, prior to either treatment, a significantly higher percentage of PA patients had an elevated LVM compared with the primary hypertension group despite similar BP levels, suggesting that aldosterone excess promotes disproportionate cardiac hypertrophy. After treatment, both the adrenalectomy and medical therapy groups showed a significant decrease in LVM (Rossi et al., 2013[51]).

## Conclusion

LVH is a hallmark finding of hypertensive heart disease. Hypertensive LVH often precedes the clinical manifestation of hypertensive heart disease and is a significant prognostic marker for an increased risk of HF and cardiovascular mortality (Bang et al., 2017[4]; Velagaleti et al., 2013[59]). Therefore, screening and diagnosis of LV remodeling is critical in patients with hypertension. ECG is a first-line diagnostic modality for detecting LVH in hypertensive patients, but traditional ECG criteria have limited sensitivity. AI-based ECG models appear to outperform traditional ECG measures in detecting LVH, and they hold promise for earlier detection of LV remodeling. Echocardiography is the most commonly used imaging modality for detailed evaluation of LV geometry, while CMR can provide improved diagnostic accuracy and tissue characterization in select patients. Biomarkers, such as high-sensitivity troponin and natriuretic peptides, can be helpful for predicting future cardiovascular morbidity among individuals with asymptomatic hypertensive LVH. Because of the association between LVH and HF, preventing LVH and the progression from LVH to clinical HF is crucial to improving patient outcomes and reducing mortality.

## Declaration

### Artificial Intelligence (AI) - assisted technology

The authors used Grammarly to assist with proofreading, grammar correction, and improving the overall readability of this manuscript. The authors reviewed and edited all suggestions and take full responsibility for the final content.

### Conflict of interest

The authors declare that they have no relevant disclosures.

## Figures and Tables

**Figure 1:**
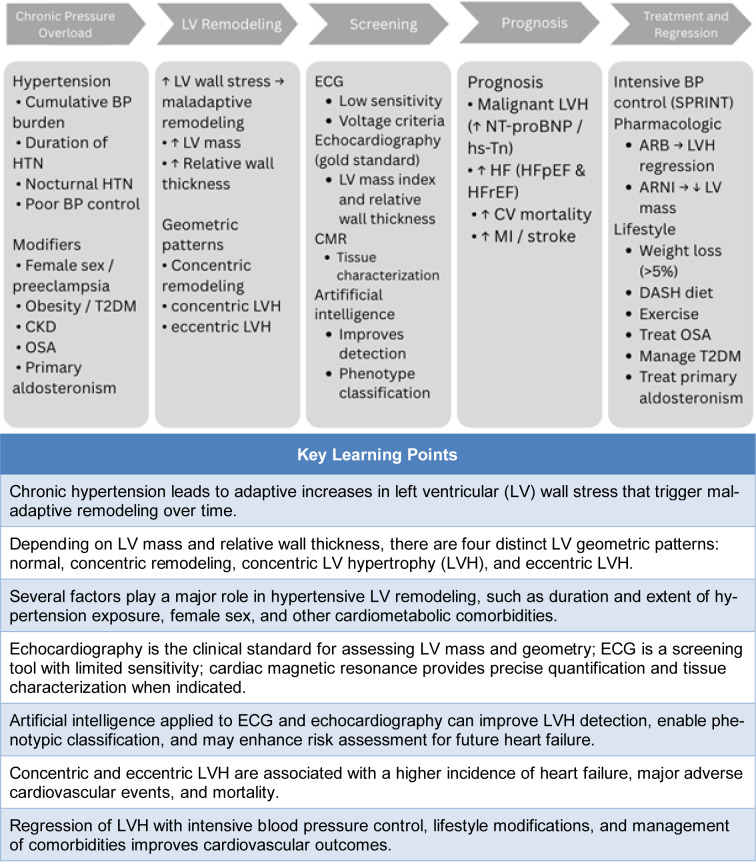
Graphical abstract: Mechanisms, diagnosis, and management of hypertensive LV remodeling. BP, Blood Pressure; HTN, Hypertension; T2DM, Type 2 Diabetes Mellitus; CKD, Chronic Kidney Disease; OSA, Obstructive Sleep Apnea; LV, Left Ventricle; LVH, Left Ventricular Hypertrophy; ECG, Electrocardiogram; CMR, Cardiac Magnetic Resonance Imaging; NT-proBNP, Amino-Terminal Pro-B-type Natriuretic Peptide; hs-Tn, high-sensitivity cardiac troponin T; HF, Heart Failure; HFpEF, Heart Failure with Preserved Ejection Fraction; HFrEF, Heart Failure with Reduced Ejection Fraction; CV, Cardiovascular; MI, Myocardial Infarction; SPRINT, Systolic Blood Pressure Intervention Trial; ARB, Angiotensin II Receptor Blocker; ARNI, Angiotensin-Receptor Blocker-Neprilysin Inhibitors; DASH, Dietary Approaches to Stop Hypertension

**Figure 2:**
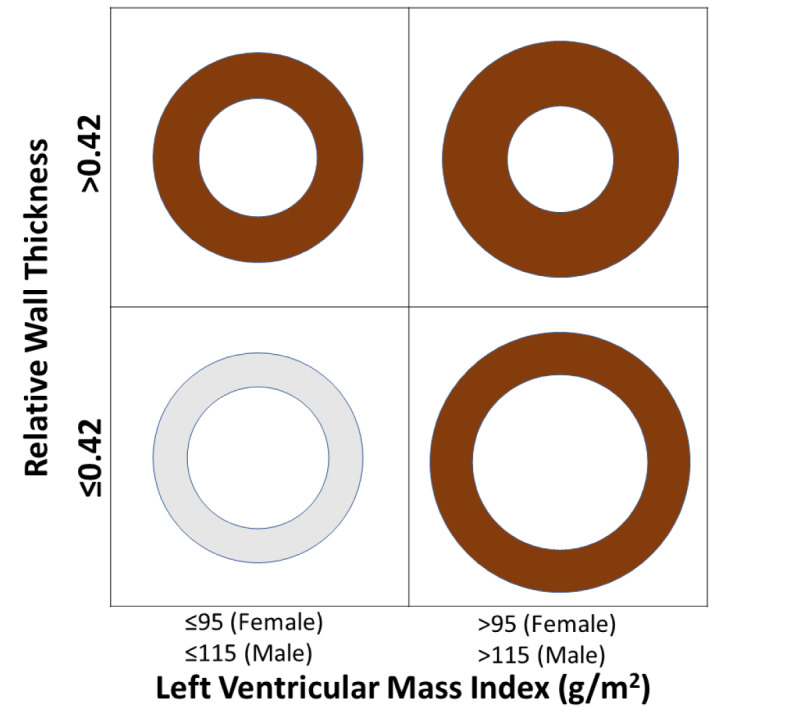
Left ventricular geometry patterns

**Figure 3:**
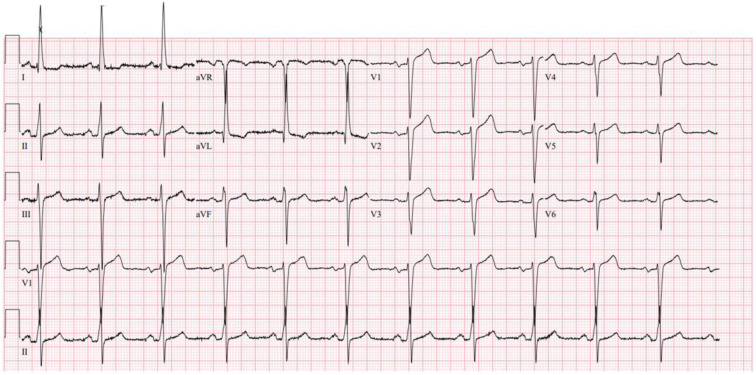
Electrocardiographic evaluation of left ventricular hypertrophy; 60 y/o female with hypertension and aortic stenosis; Sokolow-Lyon criteria (SV1 + RV5/RV6), (21 + 4 = 25 mm, not meeting criteria); Cornell criteria (RaVL + SV3), (21 + 12 = 33 mm, meeting criteria); Peguero-Lo-Presti criteria (deepest S wave in any lead [SD] + S wave in lead V4 [SV4]), (20[SV1] + 11 = 31 mm, meeting criteria)

**Figure 4:**
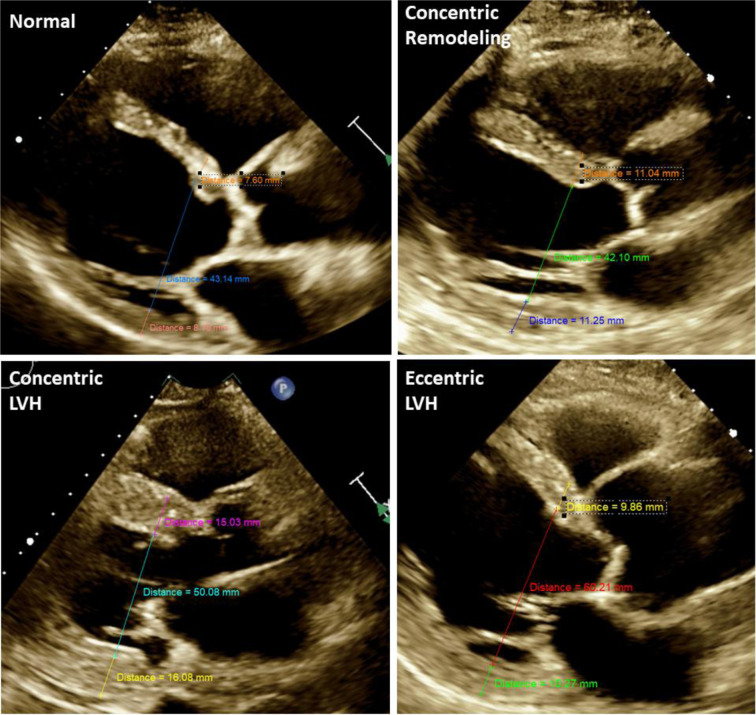
Echocardiographic characterization of the left ventricular geometry using linear measurements; Normal: 65 y/o female with BSA of 1.6 m^2^; IVSd = 8 mm, LVIDd = 43 mm, PWTd = 8 mm, LVM/BSA = 66 g/m^2^, RWT = 0.37; Concentric remodeling: 60 y/o female with BSA of 2.0 m^2^; IVSd = 11 mm, LVIDd = 42 mm, PWTd = 11 mm, LVM/BSA = 79 g/m^2^, RWT = 0.52; Concentric LVH: 49 y/o female with BSA of 2.0 m^2^; IVSd = 15 mm, LVIDd = 50 mm, PWTd = 16 mm, LVM/BSA = 169 g/m^2^, RWT = 0.64; Eccentric LVH: 72 y/o female with BSA of 2.0 m^2^; IVSd = 10 mm, LVIDd = 60 mm, PWTd = 10 mm, LVM/BSA = 123 g/m^2^, RWT = 0.33. BSA, body-surface area; IVSd, interventricular septal diameter (end-diastole); LVIDd, left ventricular internal diameter (end-diastole); LVM, left ventricular mass; PWTd, posterior wall thickness (end-diastole); RWT, relative-wall thickness
